# Micro/nanostructural properties of peri-implant jaw bones: a human cadaver study

**DOI:** 10.1186/s40729-022-00417-3

**Published:** 2022-04-11

**Authors:** Kazuto Koresawa, Satoru Matsunaga, Atsuhiko Hikita, Hajime Okudera, Akira Yamaguchi, Yasutomo Yajima, Shinichi Abe

**Affiliations:** 1 Department of Anatomy, Tokyo Dental College, 2-9-18 Kandamisaki-cho, Chiyoda-ku, Tokyo, 101-0061 Japan; 2 Oral Health Science Center, Tokyo Dental College, 2-9-18 Kandamisaki-cho, Chiyoda-ku, Tokyo, 101-0061 Japan; 3 Department of Cell and Tissue Engineering (Fujisoft), Graduate School of Medicine, The University of Tokyo, 7-3-1, Hongo, Bunkyo-ku, Tokyo, 113-8655 Japan; 4 Tokyo Plastic Dental Society, 2-26-2 Ohji, Kita-ku, Tokyo, 114-0002 Japan; 5 Department of Oral and Maxillofacial Implantology, Tokyo Dental College, 2-9-18 Kandamisaki-cho, Chiyoda-ku, Tokyo, 101-0061 Japan

**Keywords:** Peri-implant bone, Osteon, Second-harmonic generation imaging, Biological apatite (BAp) orientation

## Abstract

**Purpose:**

Many points concerning the structure of osseointegration and the surrounding jaw bone remain unclear, and its optimal histological form has yet to be identified. The aim of this study was to clarify the structural characteristics of peri-implant jaw bone on the micro- and nano-scales by quantitatively evaluating bone quality.

**Methods:**

Five samples of human mandibular bone containing dental implants and one dentate sample that had been in place for some years while the donors were still alive were collected. Bulk staining was performed, and 100-μm-thick polished specimens were prepared. The osteon distributions in peri-implant bone and mandibular cortical bone were measured, after which alignment analysis of biological apatite (BAp) crystallites and anisotropy analysis of collagen fiber orientation using second-harmonic generation imaging were carried out.

**Results:**

Osteons in the vicinity of the implant body ran parallel to it. In the cortical bone at the base of the mandible, however, most osteons were oriented mesiodistally. The preferential alignment of BAp crystallites was generally consistent with osteon orientation. The orientation of collagen fibers in peri-implant jaw bone resembled the concentric rings seen in normal cortical bone, but there were also fibers that ran orthogonally across these concentric fibers.

**Conclusions:**

These results suggest that the mechanical strain imposed by implants causes the growth of cortical bone-like bone in areas that would normally consist of cancellous bone around the implants, and that its structural characteristics are optimized for the load environment of the peri-implant jaw bone.

## Background

Dental implants have become an essential aspect of dental treatment for loss of teeth worldwide in recent years, and implants of a wide variety of forms and materials have been developed. Most are designed on the principle of osseointegrated implants introduced into clinical use by Brånemark et al*.* in 1965 [1]. Osseointegrated implants are directly bonded to the jaw bone by osseointegration, defined as “the direct structural and functional connection between living bone and the surface of a load-bearing artificial implant” [2, 3]. In clinical practice, the success of implant surgery with achievement of osseointegration is determined by using computed tomography (CT) or other diagnostic imaging to confirm the extent of contact, the contact rate, between the implant and jaw bone or by testing the mobility of the implant. These methods are used because it is currently difficult to determine whether good osseointegration has been achieved in terms of the volume or thickness of the lamellar bone formed. The results of optical microscopy studies suggest that the contact rate between implant and bone should be 50–70%, with too high a contact rate also problematic [4]. This is because perfusion is essential for homeostatic maintenance of peri-implant bone, and poorly vascularized lamellar bone may thus not be ideal [5, 6]. Many points concerning the structure of osseointegration and the surrounding jaw bone remain unclear, and its optimum histological form has yet to be identified. In addition, there are no reports of human jaw bone, since reports were mainly of animal experiments that functioned for a long time [7, 8].

Attention is now focused on attempts to assess the mechanical function of the newly formed bone using analytical techniques derived from materials engineering. Since the National Institutes of Health (NIH) Consensus Development Panel took place in 2000 [9], the bone strength of diseased or new bone can be predicted with a high degree of accuracy by the quantitative evaluation of bone quality, as well as bone mass. The structural characteristics of bone on the micro- and nano-scales are strongly affected by localized stress. In particular, collagen fibers and biological apatite (BAp) crystallites affect bone quality in terms of resistance to tensile stress and compressive stress, respectively [10]. BAp orientation indirectly reflects the running of collagen fibers and is a hexagonal ionic crystal with strong anisotropy. The c-axis coincides with the long-axis direction of the crystal and extends in this direction [11]. Using a microbeam X-ray diffraction system, the characteristic preferential orientation of the c-axis according to the distribution of in vivo stress has been clarified in the rabbit ulna and skull [10]. C-axis orientation responds to and changes external stress sensitively, and is strongly related to mechanical function, and it is attracting attention as an important bone quality index in the nano-order that directly reflects bone strength. Anisotropy analysis of these two factors enables an examination of the mechanical environment in terms of qualitative parameters of the bone formed around implants [12–15].

The aim of this study was to clarify the structural characteristics of peri-implant jaw bone on the micro- and nano-scales by quantitatively evaluating bone quality.

## Methods

### Specimens

**Fig. 1:**
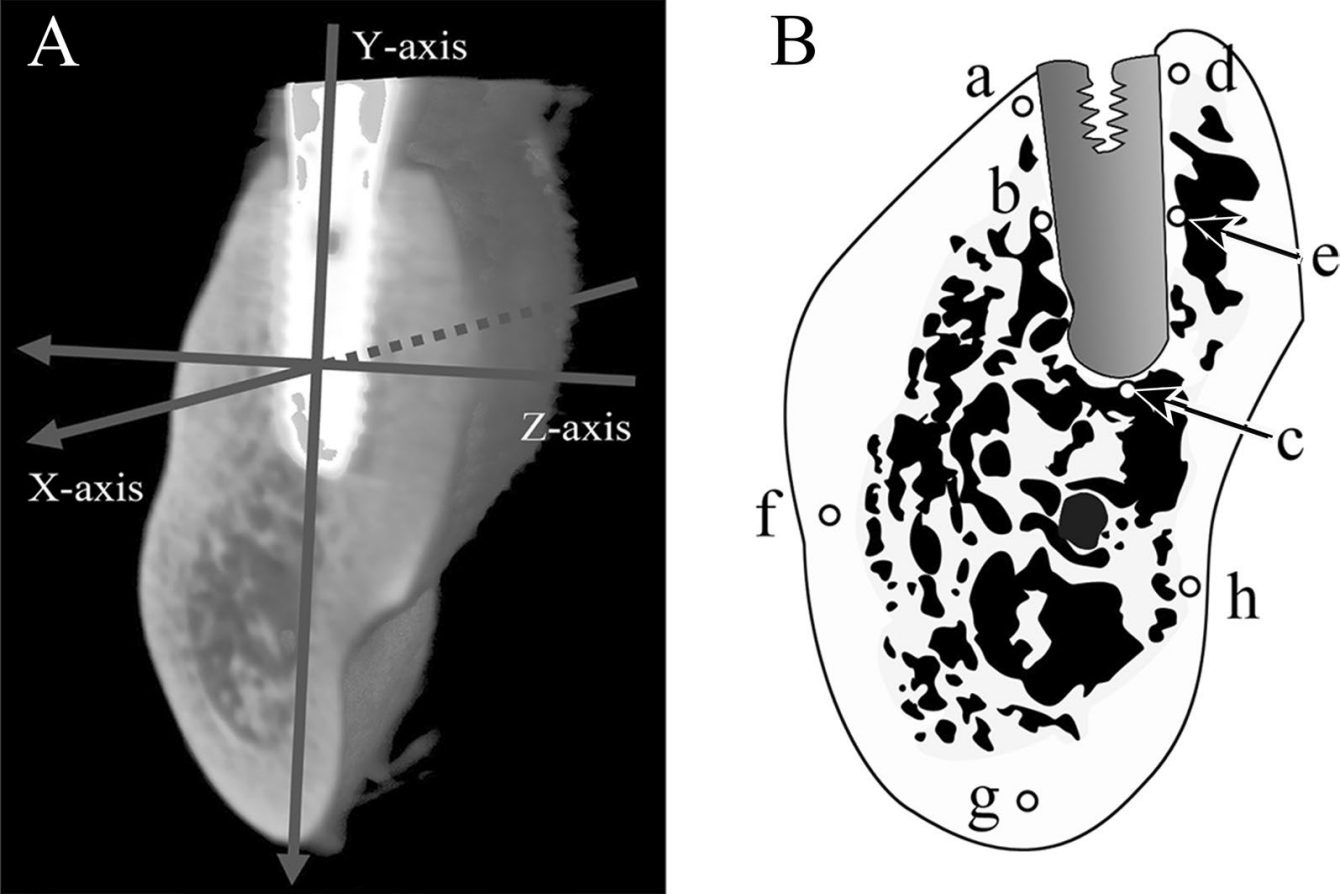
Sagittal cross-sections at each measurement location Mandibular coordinate axis and sagittal cross-sections at each measurement location. **A** The *X*-axis indicates mesiodistal axis. The *Y*-axis indicates the implant body axis. The *Z*-axis indicates the buccolingual axis. **B** (a) Implant neck, buccal side; (b) central part of the implant, buccal side; (c) implant apex; (d) implant neck, lingual side; (e) central part of the implant, lingual side; (f) cortical bone at the base of the mandible, buccal side; (g) cortical bone at the base of the mandible; (h) cortical bone at the base of the mandible, lingual side

Jaw bones containing implants were obtained from five cadavers of Japanese adults (mean age: 73.6 years, SD ± 15.8 years) in the possession of the Department of Anatomy of Tokyo Dental College. The samples were selected from those with no implant collar exposed before life, and the ones in which the implants were functioning normally were used. Table 1 shows the details of the peri-implant jaw bones used. The samples were resected en bloc as frontal sections containing the implant and its surrounding jaw bone, taken from the first premolar region in the anterior part and the first molar region in the posterior part of the mandible. The mesiodistal direction was designated the *X*-axis of the sample, the long axis of the implant body the *Y*-axis, and the buccolingual direction the *Z*-axis (Fig. 1A).

**Table 1:** Details of the cadavers’ age, period of implant use, sex, and dental implants’ condition

Specimen	a	b	c	d	e
Age, y	88	82	73	Unknown	52
Period of use (years)	22	12	7	Unknown	Unknown
Sex	Male	Male	Female	Male	Male
Type	Cylinder	Cylinder	Cylinder	Screw	Screw
Implant size (mm)	φ4.1 × 10	φ4.1 × 10	φ4.1 × 10	φ4.1 × 10	φ4.1 × 10
Part (FDI)	46	46	36	46	46
Mesial con	Tooth	Implant	Tooth	Tooth	Tooth
Distal con	Implant	Implant	Implant	Implant	Implant

This study was approved by the Ethics Committee of Tokyo Dental College (approval number 783) and the Research Ethics Committee of the University of Tokyo Graduate School of Medicine and School of Medicine (approval number 11764).

### Histological examination of peri-implant jaw bone

The samples were fixed in 10% formalin and dehydrated in 70% ethanol. Bulk staining was then performed, and the samples were embedded in methyl methacrylate resin. The samples were sectioned along the longitudinal axis of the implant (*Y*-axis) using an SP1600 saw microtome (Leica Biosystems, Nussloch, Germany) with a 300-μm blade and polished with waterproof sanding paper (#400 to #800 to #1200) to produce 100-μm-thick polished specimens. Each parameter was measured at the eight locations shown in Fig. 1. The prepared polished specimens were examined using a universal optical microscope (UPM Axiophot 2, Carl Zeiss, Oberkochen, Germany) to observe their morphology, the presence or absence of microcracks or remodeling, focusing on the peri-implant jaw bone.

### Anisotropy analysis of collagen fiber orientation

Second-harmonic generation (SHG) imaging was carried out using a high-speed multiphoton confocal microscope (A1R + MP, Nikon, Tokyo, Japan) with a laser oscillator (wavelength 690–1040 nm, repetition rate 80 MHz, and pulse width 70 fs) (Mai Tai eHP, Spectra-Physics, Andover, MA) and a water-immersion objective lens (numerical aperture 1.1) (CFI75 Apo 25 × W MP, Nikon). The excitation wavelength used for collagen fiber observations was 880 nm. Image acquisition, orthogonal view processing, and trimming were carried out using NIS-Elements ver. 4.0 (Nikon). As shown in the legends to the corresponding figures, the look-up tables for this software were used to adjust the brightness and contrast of a number of images on the basis of the shared parameters of associated images. From the image obtained, a square region of 200 µm × 200 µm at the implant neck (a, d), a region at the central part (b, e) and a region at the implant apex (c) were extracted as the regions of interest (Fig. 1B). High-precision image analysis software (Imaris8.4, Bitplane AG, Zürich, Switzerland) was used to trace and measure the angles of the collagen fiber bundles.

### BAp crystal orientation

Quantitative analysis of BAp crystallite alignment was carried out using Cu-Kα by a microbeam X-ray diffraction system (RINT RAPID II, Rigaku, Tokyo, Japan) with two optical systems: a reflection system and a transmission system. The tube voltage was set at 40 kV and the tube current at 30 mA for all measurements. A collimator 100 μm in diameter was used for the incident beam. First, the *X*-axis was measured using the reflection optical system of the diffraction system. In this process, the diffraction X-ray beam was detected with a curved positron-sensitive proportional counter. The *Y*- and *Z*-axes were measured using the transmission optical system of the diffraction system. The measurement conditions were those used by Nakano [10]. When using the transmission diffraction system, the diffraction beam traced a ring shape on an imaging plate (IP). Quantitative evaluation of BAp crystallite alignment was carried out by scanning this IP using detection software (2DP, Rigaku) and calculating the intensity ratio of the two diffraction peaks at (002) and (310).

### Statistical analysis

After each of the measurement fields had been calculated, the Kruskal–Wallis test, one-way analysis of variance, and Tukey’s multiple comparison test were carried out using SPSS (version 23.0, IBM Corporation, Chicago, IL) statistical software, with *p* < 0.05 regarded as significant.

## Results

### Histological examination

**Fig. 2:**
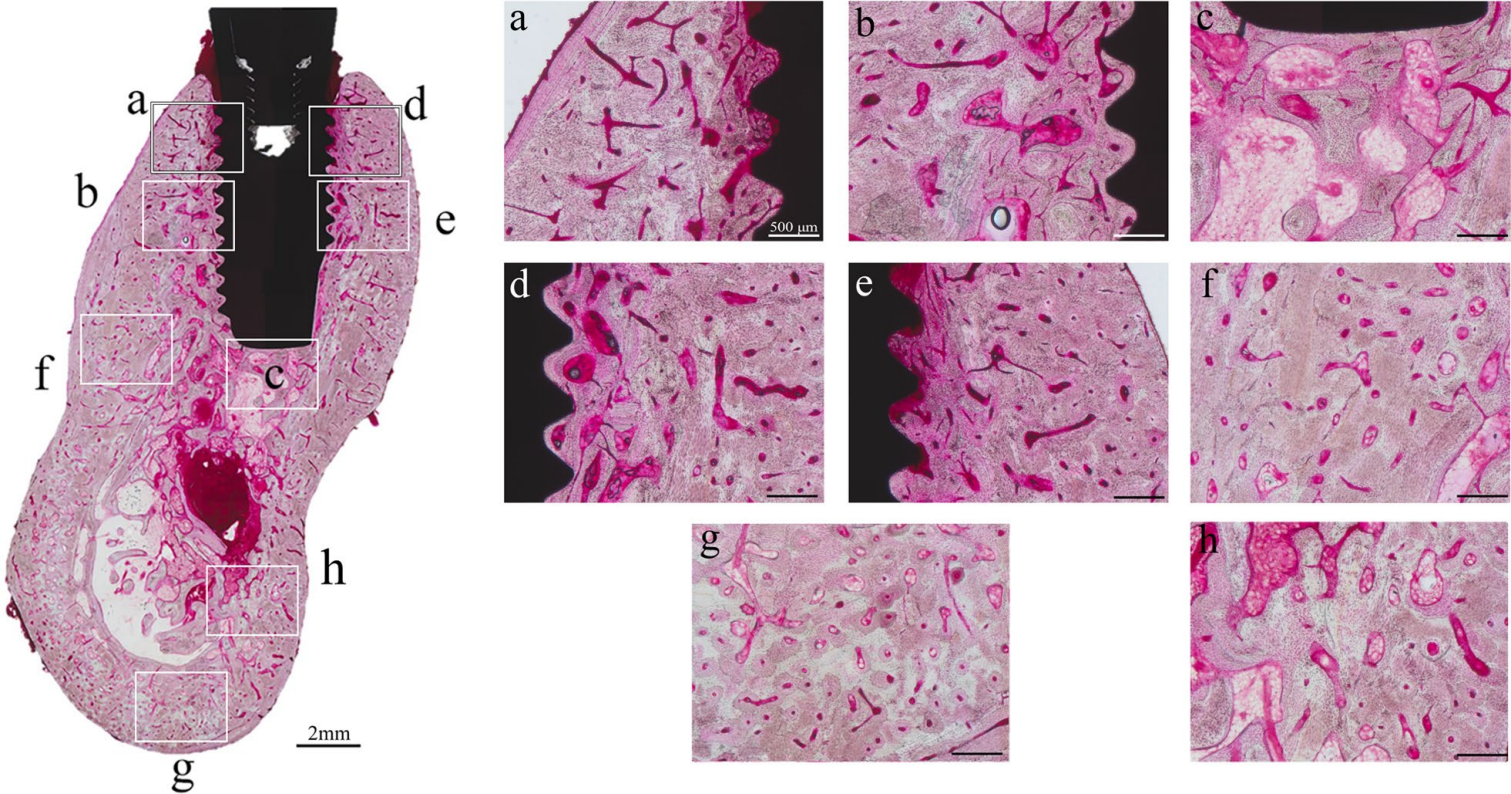
Bulk staining images of peri-implant jaw bone at each measurement location. Rich canals for blood vessels in the vicinity of the implant body run in various directions. In the cortical bone at the base of the mandible, but most osteons are oriented mesiodistally. Osteons have been generated in large quantities in areas that would normally consist of cancellous bone around the implants

Figure 2 shows bulk-stained peri-implant jaw bone. The cortical bone at the base of the mandible contained a number of microfractures (Fig. 2g), but these were almost absent from cylinder and screw type peri-implant jaw bone. Osteonal bone was present not only in normal cortical bone, but had also been generated in large quantities in areas that would normally consist of cancellous bone around the implants. In the cortical bone at the base of the mandible, the osteons were mainly aligned mesiodistally and were elliptical in form.

The bone at the implant–bone interface contained osteonal structures running parallel to the long axis of the implant, with mesiodistally aligned osteons adjacent to them. Osteonal bone growing in areas that would normally consist of cancellous bone had a structure similar to that of cortical bone and was continuous with alveolar cortical bone in many places. Large amounts of osteonal bone were also present at the implant tip, along with elliptical structures with their long axis oriented buccolingually.

### Orientation anisotropy of collagen fiber bundles

**Fig. 3:**
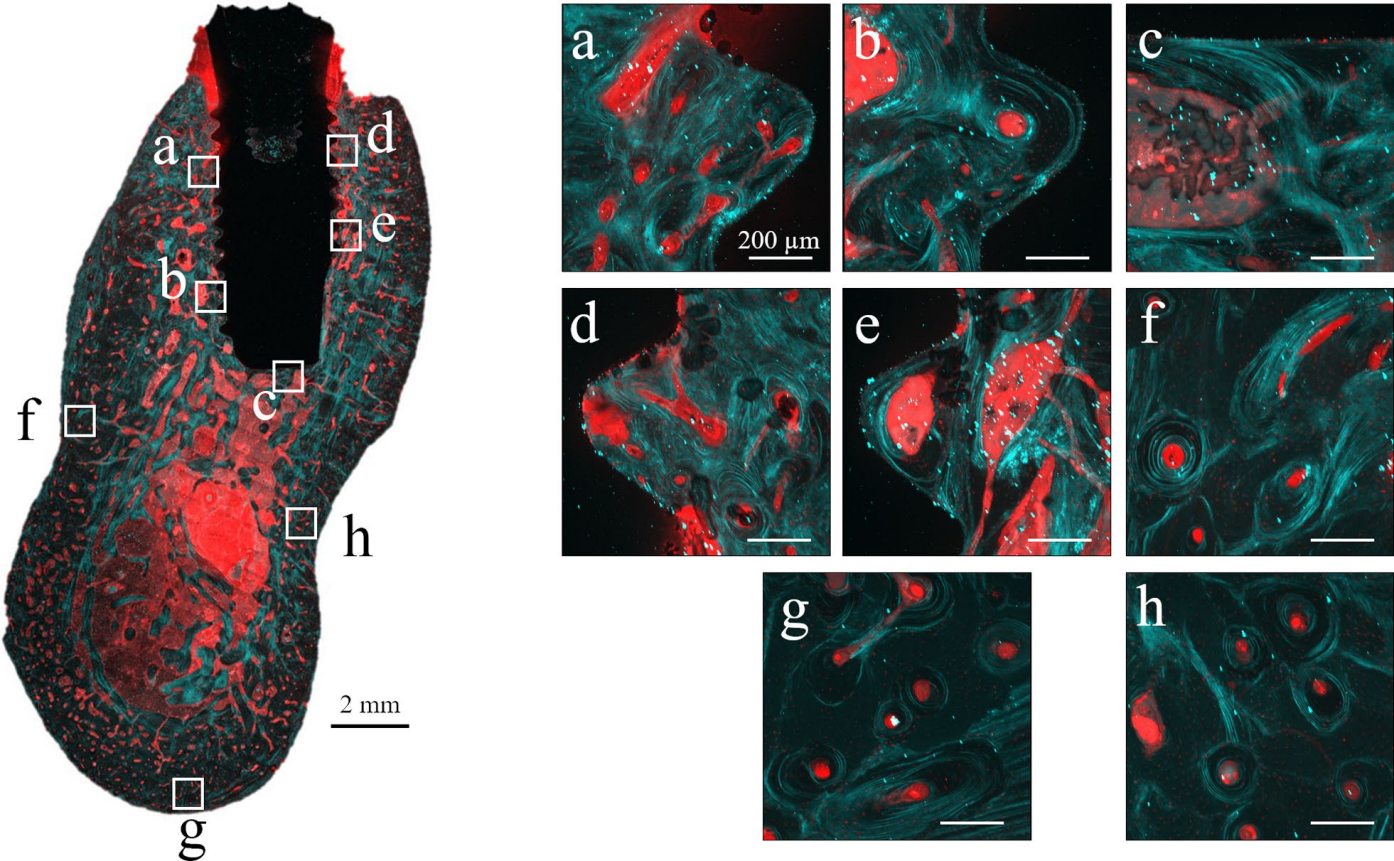
Analysis of the orientation anisotropy of collagen fibers at each measurement location using SHG imaging. Collagen fibers with thicknesses measuring ≥ 345 nm are shown in green, and other structures are indicated in red

**Fig. 4:**
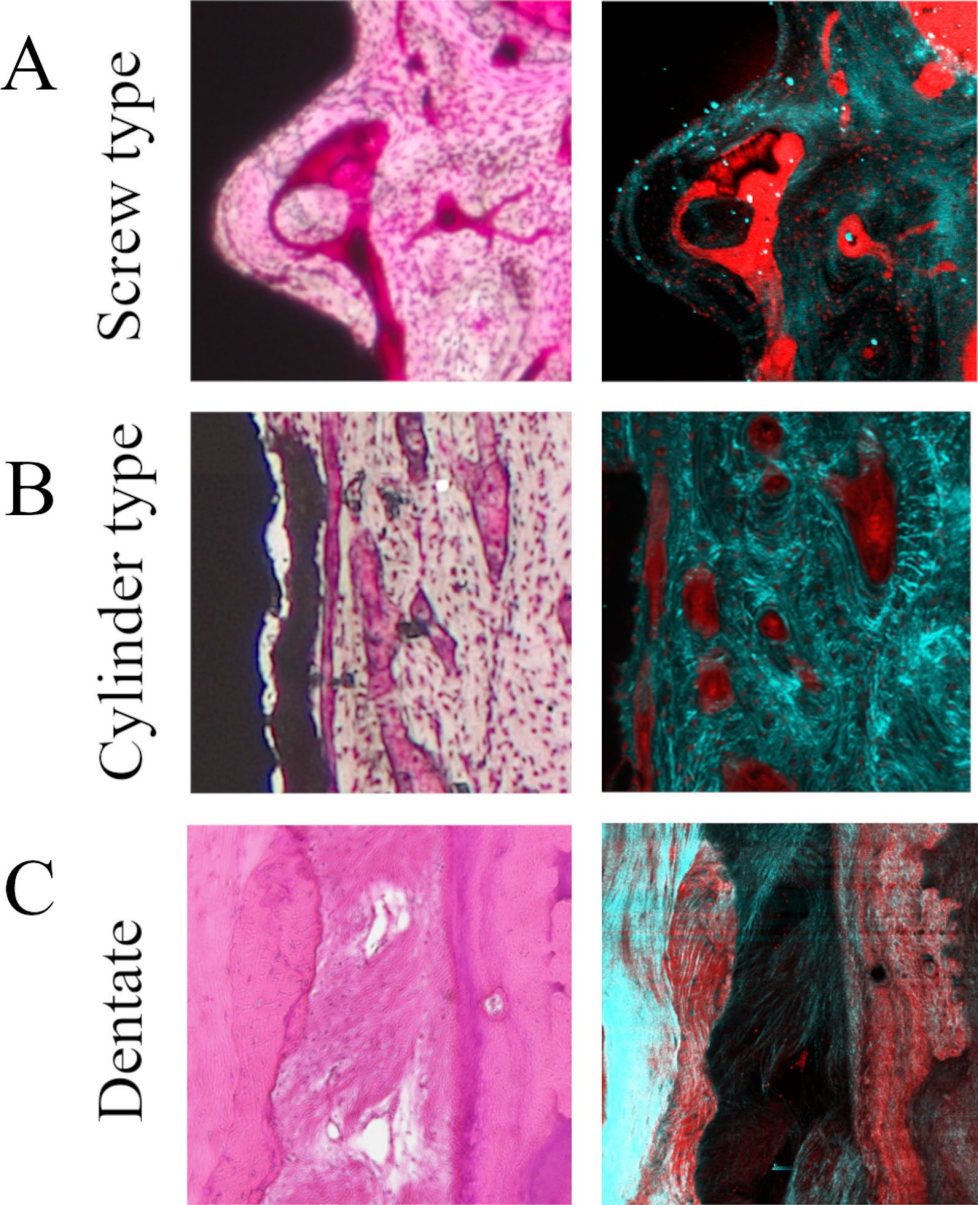
Bulk-stained osteonal bone in the vicinity of the implant and collagen fibers running in the interior. **A** Screw type. **B** Cylinder type. **C** Dentate

**Fig. 5:**
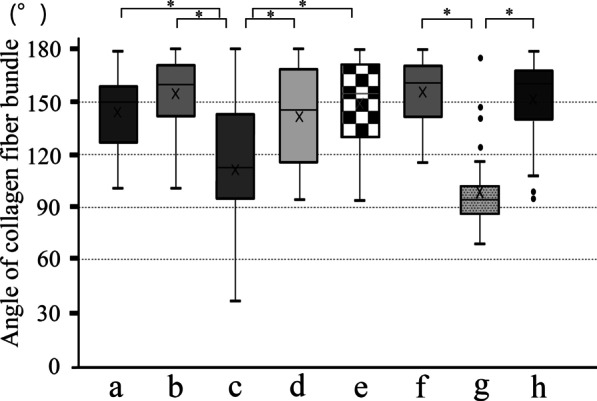
Angles of orientation of collagen fibers at each location. Box-and-whisker plot of the angle of the collagen fiber bundle with respect to the *Y*-axis. The vertical axis shows the number of collagen fibers, and the horizontal axis shows the angle with respect to the axis of the implant body set as 0°

Figure 3 shows SHG imaging of peri-implant jaw bone. SHG imaging confirmed collagen fiber bundles with distinctly different running in the peri-implant bone and normal bone (Fig. 3a–e). Not only were abundant collagen fiber bundles resembling osteons apparent around the cylinder and screw type implant, but these collagen fiber bundles were running in a variety of different directions (Fig. 4A, B). On the other hand, in the dentate specimen collagen fiber bundles were found to run regularly from the alveolar bone toward the cementum (Fig. 4C). Cortical bone at the base of the mandible contained concentric rings of collagen fibers with Haversian canals at their center (Fig. 3f–h). The angles of the courses of the collagen fiber bundles in each region are shown as box-and-whisker plots in Fig. 5. In peri-implant bone, the implant apex region (c) was the only area in which collagen fiber bundles ran perpendicular to the direction of implant insertion, and in all the other areas of the implant neck region and the central part of the implant body region (a, b, d, e), they ran parallel to the direction of implant insertion.

### BAp crystallite alignment

**Fig. 6:**
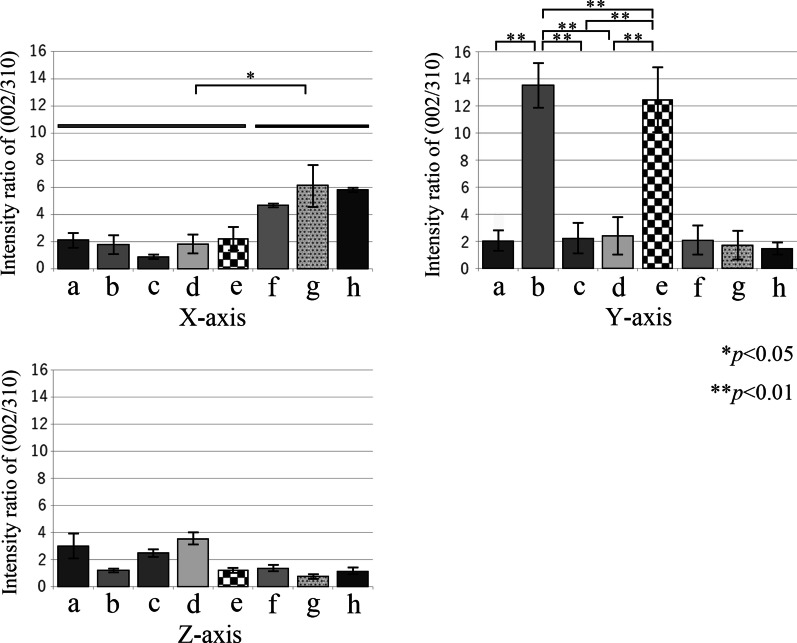
Orientation of BAp *c*-axis in each measurement axis. The vertical axis shows the X-ray diffraction intensity ratio calculated from the (002)/(310) peaks, and the horizontal axis shows the groups. **a** *X*-axis (mesiodistal axis) direction. **b** *Y*-axis (the long axis of the implant body) direction. **c** *Z*-axis buccolingual axis direction

Figure 6 shows the X-ray diffraction intensity ratios calculated for the three axes for use in the quantitative evaluation of bone quality in the peri-implant jaw bone. The intensity ratio of hydroxyapatite powder was 1.04 for the reflection system and 3.13 for the transmission system. The X-ray diffraction intensity ratios on the *X* -, *Y* -, and *Z*-axes for each category (a-h) were evaluated using the mean values for measurement.

At the base of the mandible (f–h), the preferential alignment was uniaxial in the *X*-axis direction. In addition, in peri-implant bone (a-e), the X-ray diffraction intensity ratio in the *X*-axis orientation was significantly lower in all regions than it was at the base of the mandible (f, g, h) (*p* < 0.05). However, there was no significant difference between peri-implant bone in any of these regions. A strong preferential uniaxial orientation in the *Y*-axis direction was evident in the central part of the implant body region (b, e), but in the implant neck and implant apex regions (a, c, d), no BAp crystallite alignment in the *Y*-axis direction was evident. In the *Z*-axis direction, there was a tendency for the X-ray diffraction intensity ratio to be comparatively higher in the implant neck region.

## Discussion

The clinical definition of osseointegration is not necessarily consistent with the definition used in basic research and remains the subject of active debate. Clinically, osseointegration is described as “the state of direct contact between the implant body surface and bone” [16]. However, subsequent studies have found that proteoglycans around 50 nm in size are sandwiched between implants and jaw bone, and the implant body is thus not in “direct” contact with the jaw bone [17]. Further, the state of osseointegration refers only to the condition of the bone–implant interface, and studies of the surrounding bone have varied widely. In terms of bone morphology at the implant–bone interface, Tonino et al*.* reported that it is composed of lamellar bone containing regularly arranged collagen fibers [18–22]. Iezzi et al*.* conducted histological and morphological observations of human peri-implant jaw bones removed from eight individuals for various different reasons and reported that osteonal bone was present, in addition to lamellar bone around the implant body [23]. Data from studies by Kiani et al*.* and Feng et al*.* also showed large amounts of peri-implant osteonal bone structure [24, 25]. Al-Hamdan et al*.* reported that osteonal bone structure was recognized on the side (outer side) of the implant–bone interface [26]. They named this structure a “compacted-cancellous structure” and reported that it indicated that good osseointegration had been achieved. In the present study, the osteonal bone structure approximately 250 µm from the implant body was also observed. Peri-implant jaw bone requires a rich supply of oxygen and nutrients, meaning that osteons with Haversian canals or Volkmann’s canals that enclose blood vessels grow more rapidly than lamellar bone, which has a poor blood supply. Regardless of implant shape, osteons adjacent to the implant body were oriented parallel to it, but those comparatively further away tended to be oriented mesiodistally. This may have been because the insertion of the implant body blocked off vascularization in the mesiodistal direction, restricting the orientation in which Haversian canals could form.

Recently, advances in bioimaging have enabled SHG imaging nonlinear optical microscopy, allowing collagen fibers to be selectively visualized [27, 28]. The observations of cortical bone in the implant neck region using this new technology in the present study showed the presence of collagen fibers within the lamellar bone that were orthogonal to the implant body. This orientation anisotropy may have been generated to resist the depression of the implant that occurs during occlusion. The collagen fibers in osteons of the cortical bone formed concentric rings surrounding Haversian canals at their centers. However, collagen fibers in the newly grown osteons in the vicinity of the implant body not only formed concentric rings, but also ran orthogonally to these rings. In the peri-implant bone overall, however, with the exception of the orientation anisotropy of collagen fiber bundles in the horizontal direction at the implant apex region, all collagen bundles ran orthogonally to the implant orientation. In the dentate specimens, the stress applied to the tooth is relieved through the periodontal ligament. Therefore, it runs in a certain direction from the alveolar bone toward the cementum. On the other hand, in the case of implants, the result of the optimization of bone by the acquisition of orientation anisotropy of collagen fibers due to the direct connection between the implant body and the jaw bone to transmit and cushion load, without the mediation of the periodontal ligament. As a result of BAp crystallite alignment analysis, it has been reported that BAp crystallite arrangement in the first molar of human mandibular cortical bone was low in the alveolar region. However, it was also shown that the implant had a very weak biaxial orientation in both the mesiodistal direction and the buccal tongue direction of the implant neck region, but showed a strong uniaxial orientation in the occlusal direction in the central part of the implant body [29]. These results suggest that the nanostructural characteristics are optimized to support and cushion the horizontal load imposed on the neck of the implant and the vertical load transmitted to the inside of the jaw bone at the central part of the implant body.

## Conclusions

The present results suggest that, even after osteointegration has been achieved, remodeling occurs repeatedly in the peri-implant jaw bone in a constant process of optimization to the new mechanical environment created by implant insertion. As a result, after implant insertion, new osteonal bone, which contains vessels for blood supply and is also comparatively strong, grows within the mandible in areas that would normally consist of cancellous bone in dentulous mandibles, where it plays an important biomechanical role.

## Data Availability

The datasets used and analyzed during the current study are available from the corresponding author on reasonable request.

## Abbreviations

BAp: Biological apatite
CT: Computed tomography
NIH: National Institutes of Health
SHG: Second-harmonic generation
IP: Imaging plate
